# Validation of the Zulfiqar Frailty Scale (ZFS): A New Tool for General Practitioners

**DOI:** 10.3390/medicines8090052

**Published:** 2021-09-04

**Authors:** Abrar-Ahmad Zulfiqar

**Affiliations:** Service de Médecine Interne, Diabète et Maladies Métaboliques de la Clinique Médicale B, Hôpitaux Universitaires de Strasbourg et Equipe EA 3072 “Mitochondrie, Stress Oxydant et Protection Musculaire”, Faculté de Médecine—Université de Strasbourg, 67000 Strasbourg, France; abzulfiqar@gmail.com

**Keywords:** ZULFIQAR Frailty Scale (ZFS), modified SEGA scale grid A, primary care, prevention, elderly subjects

## Abstract

Introduction: The early detection of frailty, a frequent transient state that can be reversible in the elderly and is responsible for significant morbidity and mortality, helps prevent complications from it. Objective: To evaluate the performance of the “ZFS” tool to screen for frailty as defined SEGA scale criteria in an ambulatory population of patients at least 65 years of age. Methods: A prospective non-interventional study conducted in Alsace for a duration of six months that included patients aged 65 and over, judged to be autonomous with an ADL > 4/6. Results: In this ambulatory population of 102 patients with an average age of 76 years, frailty, according to modified SEGA criteria grid A, had a prevalence of 19.6%. Frailty, according to the “ZFS” tool, had a prevalence of 35.0%, and all of its elements except weight loss were significantly associated with frailty. Its threshold for identifying frailty is three criteria out of six. It was rapid (average completion time: 87 s), had a sensitivity of 100%, and a negative predictive value of 100%. Conclusions: The “ZFS” tool makes it possible to screen for frailty with a high level of sensitivity and a negative predictive value.

## 1. Introduction

General practitioners, in their role of prevention and screening, are key players in detecting risky lifestyle habits. Nevertheless, frailty remains difficult to identify in primary care due to the multitude of definitions and existing diagnostic tools. Moreover, their use is not systematically adapted to the liberal mode of exercise.

Currently, no scale is valid to screen for frailty, just as there is no consensus definition. They all differ in the number and nature of their criteria assessed. Certain scales seem rather adapted to the hospital environment while others are intended primarily for the general practitioner. Table 1 describes some frailty scales (non-exhaustive list).

With the aim of harmonizing professional practices and making the identification of frailty in general medicine consultations accessible, we have proposed a frailty screening tool, the Zulfiqar Frailty Scale (ZFS). This scale is made up of six elements, which are specified in Table 2.

For scores of three or more, the elderly patient was considered by our scale to be “frail.” For scores of one or two, the patient was considered “pre-frail.” For a score of 0, the patient was considered “non-frail” or “robust.”

This tool brings together six elements which, in the literature, are significantly and independently associated with a poor prognosis in terms of morbidity and mortality and which therefore fall within the definition of a marker of frailty [11,12].

- Nutritional status
- Balance and falls
- Cognitive function
- Level of sociability
- Polypharmacy

This choice of elements was based on their rapid completion time as well as on their simplicity. As such, prior training was not required.

The originator study was conducted in a general practice in Brittany, France to validate our frailty scale, as published in the journal MEDICINES MDPI [9].

## 2. Patients and Methods

### 2.1. Method

To answer our research question, a prospective study was set up. The latter was conducted at three general medicine practices in the Haut-Rhin departments, specifically in Mulhouse (Saint-Louis) and Colmar (Alsace), for a total period of six months (from 2 November 2020 to 30 April 2021).

### 2.2. Primary Objective

The objective of the study was to validate the Zulfiqar Frailty Scale (ZFS) and to analyze its concordance with the modified SEGA scale (mSEGA) part A (Short Emergency Geriatric Assessment), rated out of 26 and comprising 13 elements (link can be found at https://reseaux-sante-ca.org/IMG/pdf/grille_de_fragilite_volet_a-b_2014.pdf (accessed on 15 August 2021) [3,4,5].

We chose the modified SEGA frailty scale because the study took place in the Grand Est (France) where this scale is used, particularly with the RéGéCa network [3,4,5].

### 2.3. Inclusion Criteria

The patients needed to be 65 years of age or over, in consultation with a general practitioner, and with an ADL (Activity of Daily Living) greater than or equal to four. Patients less than 65 years old and subjects with an ADL less than four were excluded from this study. Those living in nursing homes were also excluded, as were patients unable to express themselves or give their consent.

### 2.4. Data Collected and Analyzed

Data for the study was recorded by the general practitioner during routine consultations. For each patient, the mSEGA frailty scale part A was carried out as well as the Zulfiqar Frailty Scale. The information was then anonymized before being transmitted for collection in the study.

### 2.5. Statistical Analysis

The statistics were produced using R 3.6.1 software. Qualitative variables were expressed by their numbers and percentages by the response method; quantitative variables were expressed as mean and standard deviation. A Pearson correlation matrix was used to study the correlations between elements and between the total scores of the two scales. In order to assess the properties of the Zulfiqar Frailty Scale, a comparison was made to the mSEGA scale part A with patients considered to be frail with a score of >8/26. As such, a ROC curve could be drawn, an area under the curve was calculated, and the optimal threshold was defined with sensitivity, specificity, positive and negative predictive values, and the Youden score.

### 2.6. Administrative Elements

Informed consent was obtained from all patients included in this study. From a regulatory standpoint, the study was registered with the CNIL (National Commission for Informatics and Liberties) according to the MR-004 reference methodology and with the Heath Data Hub directory. Internal Department Ethics Committee of University Hospital of Strasbourg approved this paper for publication (No. 18-10-20).

## 3. Results

### 3.1. Description of the Population

During this collection period, 102 patients over 65 years of age were included. We did not note any refusals. The characteristics of the population included are detailed in Table 3. Table 4 and Table 5 specify the characteristics of the frailty scales used (the frailty scale known as Zulfiqar or ZFS (1) and a modified version of the mSEGA scale grid A).

### 3.2. Correlation between the SEGA and Zulfiqar Frailty Scales

Pearson’s correlation coefficient (or Pearson’s r) and its 95% confidence interval was 0.81 [0.73; 0.86]. Table 6 presents the Pearson correlation matrix between the elements of the mSEGA grid A and Zulfiqar Frailty Scale (ZFS).

### 3.3. Performance and Validity of the Zulfiqar Frailty Scale

Table 7 presents a comparison of the element scores of the Zulfiqar Frailty Scale between frail and non-frail patients.

All results evaluating our screening tool against the SEGA criteria are shown in Table 8.

• Weight loss

“Weight loss of at least 5% of body weight over the past six months” had a sensitivity of 20% and a specificity of 98%. Its negative predictive value was 83%, and its positive predictive value was 67%.

Patients responding positively to this element had a four-fold risk of being frail.

• Monopodal balance

To identify frailty, the sensitivity of the “Monopodal balance less than 5 s” element was 85%, and the specificity was 54%. Its negative predictive value was 94%, and the positive predictive value was 31%.

Patients with abnormal monopodal balance were 4.84 times more likely to be frail.

• Lives alone

The sensitivity of the “Living alone at home” element was 65%, and its specificity was 61%. Its negative predictive value was 88%, and its positive predictive value was 29%. Patients living alone were 2.35 times more likely to be frail.

• Presence of aid at home

Patients with aid at home had a 12.32-fold risk of being frail compared to patients without aid.

The sensitivity of this item was 80%, and its specificity was 89%. Its negative predictive value was 95%, and its positive predictive value was 64%.

• Memory

“Memory impairment” had a sensitivity of 55% and a specificity of 71%. Its negative predictive value was 87%, and its positive predictive value was 31%.

Patients responding positively to this element had a 2.34-fold risk of being frail.

• Polymedication

Patients responding positively to this element had a risk of being frail multiplied by 3.65.

To identify frailty, the sensitivity of the “5 or more therapeutic classes” element was 75%, and the specificity was 62%. Its negative predictive value was 91%, and its positive predictive value was 33%.

**Figure 1:**
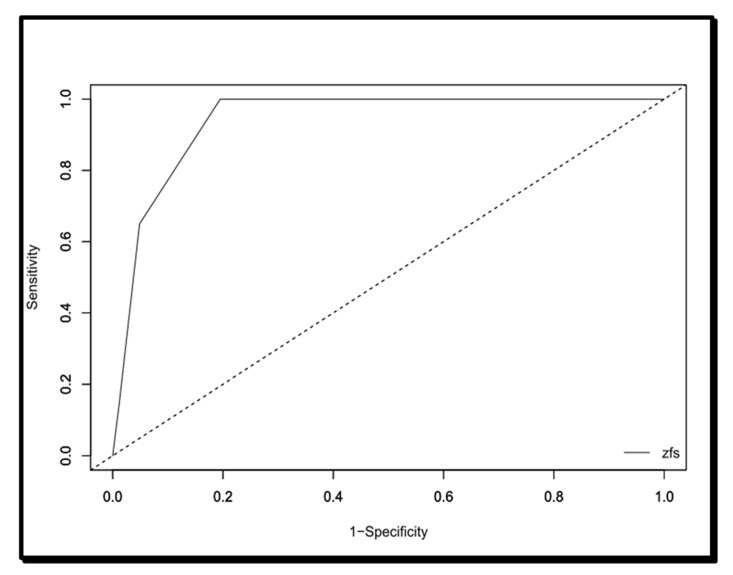
ROC curve of the Zulfiqar Frailty Scale.

Finally, the area under the curve of the Zulfiqar Fragility Scale was 0.94116, as shown in Figure 1. As shown in Table 9, the significant cut-off in terms of sensitivity, specificity, PPV, and VPN was ≥3/6.

## 4. Discussion

The challenge of screening for frailty in the elderly is growing with the current demographic changes, which will intensify in the years and decades to come. The major player in frailty screening is the general practitioner, the latter having responsibility for individual and community prevention, screening, coordination of care, and even monitoring [13].

The psychomedicosocial reflection that results from this fragility has given rise in recent years to various screening scores and scales, with the goal of managing this element within the elderly population.

The current gold standard for screening frailty in elderly patients is the Fried Phenotypic Frailty Scale [1], recommended as a first-line of treatment by the American Geriatric Society and the Haute Autorité de Santé (HAS) [14]. However, the pitfall represented by the use of a dynamometer makes this screening tool difficult to use in general medicine. In addition, this scale is only interested in frailty in the sense of sarcopenia and neglects psychosocial components.

Kenneth Rockwood’s Canadian teams conceptualized a frailty index appropriately named the Frailty Index [2,15]. Established according to a multidimensional clinical model of frailty, it is based on 70 dichotomous elements, taking into account physical elements (incontinence, cardiovascular pathology, etc.) as well as psychological and cognitive factors, and focusing on their repercussions on the activities of daily living (i.e., autonomy). This scale classifies elderly subjects into seven categories from “very robust” to “severely frail” including “in good health” or even “in good health with pathologies treated”. It considers frailty as a cumulative variable where each deficit would worsen fragility, unlike the Fried scale. However, it remains poorly suited to outpatient medicine due to time constraints, with no less than 70 elements to be completed.

In this context, other scales have emerged, particularly a scale from the Seven-Point Research Program for the Integration of Autonomy Maintenance Services (PRISMA-7) which is a scale of seven simple, self-declared elements [16,17]. This questionnaire has a high level of precision in the identification of frailty among the elderly living in communities, but tends to over-screen for frailty. In fact, Clegg et al. showed that PRISMA 7 had high sensitivity but limited specificity as a simple instrument for identifying frailty. This means that there are many false-positive test results, which limit its diagnostic test accuracy [18].

The Gérontopôle de Toulouse (GFST) frailty screening tool consists of six closed-answer questions focusing on isolation, weight loss, asthenia, walking, and memory disorders. In addition, there is a subjective question for the general practitioner: “Does your patient seem fragile to you?” [6,7,8]. The main limitation of this tool remains the subjectivity of its elements. However, it is not intended to be a scale, but simply a screening tool, making it possible to detect subjects requiring care in a day hospital.

For our study, we used the SEGA (Short Emergency Geriatric Assessment) scale, initially developed by a Belgian research team (that of Pr Schoevaerdts) to allow for rapid and early identification of the geriatric profile of elderly people admitted to emergency rooms [3]. The SEGA tool was developed from the risk factors for functional decline as described in the literature, expert opinions, and data from the DecLIC (DecLine Investigation Cohort). It studied the factors of functional decline in the emergency environment for 600 patients over 70 years old [3,4]. At the end of this process, thirteen factors were selected and grouped together in part A of the modified SEGA scale, empirically weighted in three levels (0, 1, and 2). Part B of the grid groups together categories of information, established by the various professional experiences that can facilitate or, on the contrary, make it difficult to prepare for discharge from hospital. The grid was carried out in the emergency room by a doctor or an experienced nurse with the patient and their entourage with a relatively short completion time (less than ten minutes) and was based on the patient’s situation fifteen days before their admission. The grid was neither a geriatric assessment nor a prognostic tool. It was, however, associated with certain indicators of geriatric fragility (increased length of stay, admission to geriatrics, hospital readmission within six months, mortality rates, etc.), thus enabling it to become, for each patient, a high-risk geriatric profile. It was then taken over by the RéGéCa Geriatrics Network (Champagne-Ardenne Geriatric Network) and the Reims University Hospital, after having been modified to adapt its terms and then applied to an outpatient population. In 2014, Dr. Drama’s team from the Reims University Hospital validated psychometric part A of the grid on a cohort of 167 elderly subjects living at home [4]. Tardieu et al. conducted a prospective longitudinal multi-center cohort study, hospitalized in a short stay medical ward via emergency department: the SAFES group and showed poor convergent validity (Donini instrument, Rockwood instrument, and Winograd instrument) [5].

The latest scale, developed by Dr Zulfiqar, is a multi-faceted tool, taking into account the clinical, psychological, and social dimensions of the patient. In order for it to be suitable for general medicine, it needs to be, above all, simple, efficient, and quick to conduct. In addition, it needs an appropriate level of sensitivity as well as a high negative predictive value for its validity. This was shown in the princeps study [9]. A second study, comparing the performance of the so-called Zulfiqar frailty scale with the Fried scale, confirmed the results observed in the originator study. This study was accepted in the Belgian Journal of General Medicine SSMG (Scientific Society of General Medicine) and is in the process of being published [10].

With a sensitivity of 100%, a negative predictive value of 100% and an area under the curve of 0.94, the Zulfiqar Scale showed a high level of performance in addition to relevance, since it correlates well with the SEGA scale. This study also showed, via the threshold analysis, that this scale detected the frailty of the elderly subject from the presence of three of the six elements.

Our study showed a different proportion on the frailty rate detected by the two scales. This can be explained by the non-measurement of the proportion of pre-frail subjects by the SEGA scale.

Regarding the objective of adapting to ambulatory medicine, this frailty scale seems to have several advantages. Indeed, it does not require prior training of caregivers, nor additional equipment such as a dynamometer (as is required for the Fried scale and can represent a financial impediment). The completion time was less than two minutes on average (in this study, it was an average 87 s). Time can vary from four to ten minutes to receive a modified SEGA score [3,19,20,21], as also observed in our study. This difference in treatment time is not negligible since the average duration of a consultation in general medicine is 15–16 min, or even more in the case of an elderly patient with multiple pathologies [22]. This can be a real advantage in the context of large-scale screening, especially during the quarterly prescription renewal consultation or during home visits.

Limitations: Our study sample remained small. To be validated for the purpose of studying its reproducibility, our tool must be tested in multiple general medicine practices, in urban and rural areas, and over a larger sample with many types of practitioners (doctors, nurses, physical therapists, occupational therapists). The prediction of pathological events (falls, hospitalization, and morbidity–mortality) was not studied in this research. This task will start soon.

## 5. Conclusions

The Zulfiqar Frailty Scale seems to be suitable for wide use in ambulatory medicine, whether in the office or at home, due to its simplicity and speed of execution. Ultimately, its reproducibility and its performance in predicting an unfavorable development under stress should be initiated and tested in the elderly. Further studies will be conducted in the coming months.

## Figures and Tables

**Table 1:** Frailty scales (non-exhaustive list).

Frailty Scale	Area Explored	Prevalence	Number of Items	Duration	Place of Realization
Fried [1]	Sarcopenia	11%	5	10 min	Hospital ++
Frailty Index [2]	Multidimensional	34%	70	15 min	Hospital ++
Short Emergency Geriatric Assessment (SEGA) [3,4,5]	Multidimensional	90%	24	10 min	Emergency Unit ++/Hospital ++/Primary care +/Home +
Gérontopôle Frailty Screening Tool (GFST) [6,7,8]	Multidimensional Sarcopenia	24.5%	6 + 1 (General Practitioners subjectivity)	2 min	Primary care +++ Home +++
Zulfiqar Frailty Scale (ZFS) [9,10]	Multidimensional Sarcopenia	63.7%	5	2 min	Primary care +++ Home +++
Comprehensive Geriatric Assessment (CGA)	Multidimensional All areas of geriatrics	-	MNA, ADL, IADL, MMS, TCH, miniGDS, Walk test, Vision, Audition, Polymedication, Social, Financial status	More than one hour	Hospital +++++

+: little used; ++: ofently used; +++: frequently used; +++++: exclusively used.

**Table 2:** Criteria of the Zulfiqar Frailty Scale [9,10].

Zulfiqar Frailty Scale (ZFS)		
Is there a weight loss greater than or equal to 5% in 6 months?	Yes	No
Monopod support test < 5 s?	Yes	No
Does the person live alone at home?	Yes	No
Are there home caregivers?	Yes	No
Does the person complain of memory problems?	Yes	No
Does the person have prescriptions for more than 5 therapeutic classes on his/her prescription history for less than 6 months?	Yes	No

**Table 3:** Description of the sample.

Population Characteristics (n = 102)	
Age m (sd)	76 (8)
Gender	
Female	55 (54%)
Male	47 (46%)
Weight (kgs), m (sd)	74 (15)
Height (cm), m (sd)	166 (9)
Medical history (%)	
Cardiovascular	70 (68.6)
Pulmonary	11 (10.7)
Gastrointestinal	13 (13)
Endocrine	25 (24.5)
Neurological	14 (14)
Psychiatric	15 (15)
Obesity	26 (25)
Oncological	17 (17)
Medication, m (sd)	4.3 (3.0)
Charlson score (comorbidities score), m (sd)	4.11 (1.81)
Hospitalization in the last 6 months	
Yes	10 (9.8%)
No	92 (90.2%)
Fall in the last 6 months	
Yes	17 (17%)
No	85 (83%)
ADL (/6), m (sd)	5.83 (0.35)
IADL (/4), m (sd)	7.04 (1.68)

ADL: Activity Daily Living; IADL: Instrumental Activity Daily Living.

**Table 4:** Zulfiqar Frailty Scale (ZFS).

Zulfiqar Frailty Scale (ZFS)	
	**Population Included (n = 102)**
Zulfiqar Frailty Scale (ZFS)	Yes
Does the person live alone at home?	45 (44)
Are there home caregivers?	25 (24.5)
Is there a weight loss greater than or equal to 5% in 6 months?	6 (5.9)
Monopod support test < 5 s?	55 (54)
Does the person complain of memory problems?	35 (34)
Does the person have prescriptions for more than 5 therapeutic classes on his/her prescription history for less than 6 months?	46 (45)
ZFS, m (sd)	2.1 (1.35)
Duration time, m	87 (72–108)
Classification	
Not frail	15 (15)
Pre-frail	51 (50)
Frail	36 (35)
Frailty according to ZFS	
No	15 (15)
Yes	87 (87)

**Table 5:** mSEGA scale grid A.

mSEGA Scale Grid A	
mSEGA, m (sd)	5.61 (3.11)
Classification	
Very frail (>11)	4 (4)
Not frail (<=8)	82 (80)
Frail (9–11)	16 (16)

**Table 6:** Pearson correlation matrix between Zulfiqar and SEGA elements and scores (see heatmap below).

	Age	Provenance	Medications	Mood	Perception of health	Fall 6 Months	Nutrition	Sickenesses	AIVQ	Mobility	Continence	Meals	Cognition
Weight loss	0.00	0.05	−0.11	0.16	0.06	0.21	0.90	−0.03	−0.07	0.11	−0.01	−0.02	0.06
Monopodal balance	0.48	0.33	0.15	0.07	0.19	0.17	−0.02	0.04	0.44	0.36	0.24	0.09	0.13
Live alone	0.23	0.30	0.17	0.05	0.02	0.30	0.03	0.14	0.10	0.29	0.03	−0.09	−0.11
Presence of home helpers	0.51	0.95	0.23	−0.03	0.19	0.23	0.08	0.10	0.61	0.60	0.14	−0.06	0.19
Memory loss	0.20	0.16	0.07	0.06	−0.02	−0.11	0.02	0.02	0.42	0.06	0.18	0.14	0.71
Polymedications	0.21	0.16	0.86	−0.03	0.03	0.06	−0.04	0.58	0.17	0.18	0.12	−0.09	0.14

**Table 7:** Comparison of ZFS element scores between frail and non-frail patients.

	ZFS		
Characteristics	Frail, N = 36	Not Frail, N = 66	*p*-Value
Weight loss			0.2
No	32 (89%)	64 (97%)	
Yes	4 (11%)	2 (3.0%)	
Monopod support			<0.001
No	5 (14%)	42 (64%)	
Yes	31 (86%)	24 (36%)	
Live alone at home			<0.001
No	10 (28%)	47 (71%)	
Yes	26 (72%)	19 (29%)	
Presence of aid at home			<0.001
No	14 (39%)	63 (95%)	
Yes	22 (61%)	3 (4.5%)	
Memory loss			0.007
No	17 (47%)	50 (76%)	
Yes	19 (53%)	16 (24%)	
Therapeutic classes			<0.001
No	9 (25%)	47 (71%)	
Yes	27 (75%)	19 (29%)	

**Table 8:** Summary of all ZFS tool results compared to SEGA criteria.

	SEGA			Se	Sp	PPV	NPV	RR
**Characteristics** **ZFS**	**Frail** **, N = 20**	**Not Frail** **, N = 82**	***p*** **-Value**					
Weight loss			0.013					
No	16 (80%)	80 (98%)		20%	98%	67%	83%	4.00
Yes	4 (20%)	2 (2.4%)						
Monopodal support			0.004					
No	3 (15%)	44 (54%)		85%	54%	31%	94%	4.84
Yes	17 (85%)	38 (46%)						
Live alone at home			0.065					
No	7 (35%)	50 (61%)		65%	61%	29%	88%	2.35
Yes	13 (65%)	32 (39%)						
Presence of aid at home			<0.001					
No	4 (20%)	73 (89%)		80%	89%	64%	95%	12.32
Yes	16 (80%)	9 (11%)						
Memory loss			0.056					
No	9 (45%)	58 (71%)		55%	71%	31%	87%	2.34
Yes	11 (55%)	24 (29%)						
Therapeutic classes			0.006					
N	5 (25%)	51 (62%)		75%	62%	33%	91%	3.65
O	15 (75%)	31 (38%)						

Se: sensitivity; Sp: specificity; PPV: positive predictive value; NPV: negative predictive value.

**Table 9:** Interpretation thresholds for the ROC curve.

Marker	Cutpoint	1-Sp	Se	Sp	Youden	PPV	NPV
ZFS	>0	1	100%	0%	0%	19%	
ZFS	>1	0,8171	100%	18%	18%	22%	100%
ZFS	>2	0,6098	100%	39%	39%	28%	100%
ZFS	>3	0,1951	100%	80%	80%	55%	100%
ZFS	>4	0,0488	65%	95%	60%	76%	92%
ZFS	>5	0,0122	15%	99%	14%	74%	83%

Se: sensitivity; Sp: specificity; PPV: positive predictive value; NPV: negative predictive value.

## Data Availability

The datasets used and/or analyzed during the current study are available from the corresponding author on reasonable request.
